# Application of An Improved HPLC-FL Method to Screen Serine Palmitoyl Transferase Inhibitors

**DOI:** 10.3390/molecules22071198

**Published:** 2017-07-17

**Authors:** Simone Bertini, Giuseppe Saccomanni, Sara Del Carlo, Maria Digiacomo, Claudia Gargini, Ilaria Piano, Giuseppe Matteo Campisi, Riccardo Ghidoni, Marco Macchia, Clementina Manera

**Affiliations:** 1 Dipartimento di Farmacia, Università di Pisa, Via Bonanno 6, 56126-Pisa, Italy; giuseppe.saccomanni@unipi.it (G.S); delcarlosara@gmail.com (S.D.C.); maria.digiacomo@unipi.it (M.D.); maria.gargini@unipi.it (C.G.); ilaria.piano@unipi.it (I.P.); marco.macchia@unipi.it (M.M); clementina.manera@unipi.it (C.M.); 2 Laboratorio di Biochimica e Biologia Molecolare, Dipartimento di Scienze della Salute, Via A. di Rudinì 8, 20142 Milano, Italy; giuseppe.campisi@unimi.it (G.M.C.); riccardo.ghidoni@unimi.it (R.G.)

**Keywords:** HPLC, serine palmitoyl transferase, SPT, screening, enzymatic assay

## Abstract

In this work, we reported the application and validation of an improved high-performance liquid chromatography method coupled with a fluorimetric detector (HPLC-FL) to screen the activity of two heterocyclic derivatives reported as serine palmitoyl transferase (SPT) inhibitors. The analytical conditions were optimized in terms of the derivatization procedure, chromatographic condition, extraction procedure, and method validation according to EMEA guidelines. Once fully optimized, the method was applied to assess the SPT-inhibitory activity of the above-mentioned derivatives and of the reference inhibitor myriocin. The obtained results, expressed as a percentage of residual SPT activity, were compared to those obtained with the reference radio immune assay (RIA). The good correlation between the two types of assay demonstrated that the improved HPLC-FL method is suitable for a preliminary and rapid screening of potential SPT-inhibitors.

## 1. Introduction

Serine palmitoyl transferase (SPT) is a pyridoxal-5’-phosphate (PLP) dependent enzyme that plays a crucial role in the biosynthesis of sphingolipids [1]. It catalyzes a Claisen-like condensation between L-serine and palmitoyl-CoA, producing 3-keto-dihydrosphingosine (3-KDS) [2,3]. This reaction represents the first and rate-limiting step of the de novo biosynthetic pathway of ceramide, a sphingolipid bioactive molecule involved in fundamental cell functions, such as proliferation, differentiation, and apoptosis [4,5].

Recent studies highlight a close correlation between SPT, intracellular levels of ceramide and the onset/progression of several diseases, including neurodegenerative disorders. Mutations of the gene encoding for the first SPT subunit have been recognized as the cause of the hereditary sensory neuropathy type 1 [6,7]. In brain tissue of Alzheimer’s disease patients, high levels of ceramide and of SPT have been detected [8]. It has also been demonstrated that inhibition of the latter causes a reduction of β-amyloid (main constituent of senile plaques), while the administration of ceramide produces an opposite effect [9]. Intraocular administration of myriocin, a potent SPT-inhibitor, in RD10 mutant mice, a validated model of human *retinitis pigmentosa*, has been shown to preserve the structure and function of photoreceptors [10,11]. An enhanced ceramide synthesis, shown to be involved in ischemia/reperfusion injury and myriocin treatment, could be proposed as a strategy for myocardial pharmacological postconditioning [12]. Moreover, de novo synthesized ceramide has been shown to mediate the inflammatory response induced by *Aspergillus fumigatus* infection in mice airway epithelia, and intratracheal administration of myriocin reduced inflammation and exerted antifungal activity [13]. Finally, the anti-inflammatory action of myriocin was highlighted in a mice model of cystic fibrosis [14].

Therefore, pharmacological interventions aimed at inhibiting SPT and, as a consequence, lowering intracellular levels of ceramide, may represent a new strategy for the treatment of these diseases.

Among the currently known SPT-inhibitors, the above-mentioned natural product myriocin is the most potent and selective one. It is a widely used chemical probe in research about SPT and, more in general, sphingolipids [15]. Unfortunately, the lipophilic structure of this molecule has a negative impact on its pharmacokinetic profile and therefore limits its use as a therapeutic agent.

For the above reasons, there is a growing interest towards the discovery of new molecules as SPT-inhibitors. In the meantime, a method aimed at rapidly assessing their activities would be desirable.

At present, there is a reference radiometric inhibition assay (RIA) that involves the use of a radioactive substrate: after incubation of a cell lysate with [^3^H]L-serine, palmitoyl-CoA and cofactor (PLP), a liquid–liquid extraction of the tritiated product ([^3^H]-KDS) is performed and it is quantified using a scintillation counter [16].

A few years ago, a radioactive-free method that allows quantification of 3-KDS as the direct product of SPT reaction using a high-performance liquid chromatograph equipped with a fluorimetric detector (HPLC-FL) was reported [17]. This method uses HEK 293 cell lysate an as enzyme source and involves the reduction of 3-KDS with NaBH_4_ leading to sphinganine (*erythro*- and *threo*-sphinganine) which is derivatized with *ortho*-phthalaldehyde (OPA) and 2-mercaptoethanol. The isoindole derivative thus formed is quantified using a HPLC apparatus coupled with a fluorimetric detector.

Although such a method has led to significant improvements in terms of safety, sensitivity, and reproducibility, its application to assess the activity of SPT-inhibitors has not been reported to date.

**Figure 1:**
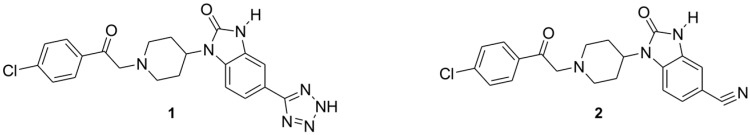
Structures of the 1,3-dihydro-2*H*-benzimidazol-2-oxo-derivatives which were screened using the radio immune assay (RIA) and HPLC-FL method.

In the present work, we describe the application of the above-mentioned HPLC-FL method, with the aim of screening the activity of some heterocyclic compounds reported as SPT-inhibitors [18]. In particular, we focused our attention on two 1,3-dihydro-2*H*-benzimidazol-2-oxo-derivatives (compounds **1** and **2**) that are described in Figure 1. For this purpose, we prepared such derivatives in our research laboratories following a different synthetic route with respect to that reported [18]. The selected compounds were tested using both the HPLC-FL assay and the reference RIA, and the results obtained were compared, highlighting a good correlation between the two methods.

It is noteworthy that rather than applying the method exactly as it was originally reported, we introduced further improvements, which consist of a slight variation in the mobile phase composition and in the extraction steps. Besides, the derivatization process of sphinganine was optimized. Furthermore, the applicability of the HPLC-FL method was assessed by testing the reference SPT-inhibitor myriocin. Finally, the method was validated by determining the accuracy and precision, the recovery and limit of quantification (LOQ), and limit of detection (LOD), in agreement with EMEA guidelines.

## 2. Results and Discussion

### 2.1. Synthesis

**Scheme 1:**
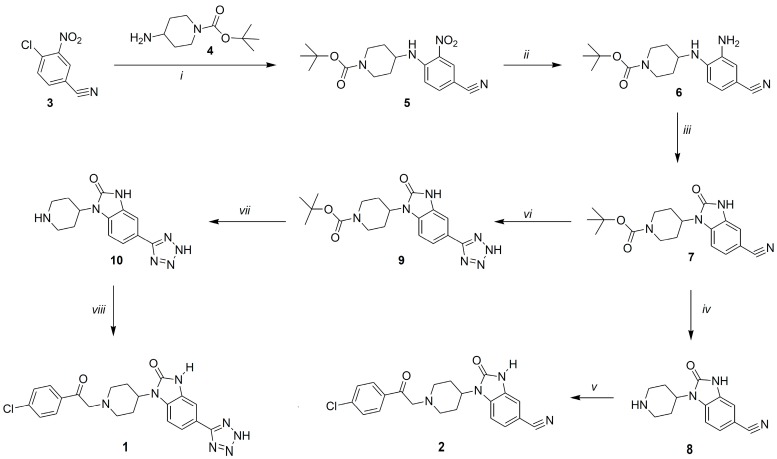
Reagents and conditions: (*i*) anhydr. DMF, DIPEA, microwave, 15 min., 130 °C, 5 bar; (*ii*) anhydr. THF, absol. EtOH, Pd-C, RT, 4 h; (*iii*) CDI, anhydr. THF, DMAP, microwave, 15 min., 130 °C, 7 bar; (*iv*) CF_3_COOH, CH_2_Cl_2_, RT, 2 h; (*v*) anhydr. DMF, Et_3_N, 2-Br-4’-chloroacetophenone, 0 °C → RT, 2 h; (*vi*) TMSN_3_, TBAF, 125 °C, 24 h; (*vii*) CF_3_COOH, CH_2_Cl_2_, RT, 2 h; (*viii*) anhydr. DMF, Et3N, 2-Br-4’-chloroacetophenone, 2 h, 0 °C → RT.

The synthesis of compound **1** and **2** is shown in Scheme 1. A variation of the synthetic approach used in the Pfizer patent to obtain the titled compounds was performed [18]. Commercial 3-chloro-4-nitrobenzonitrile **3** and commercial 4-amino-1-BOC-piperidine **4** were reacted in the presence of *N*,*N*-diisopropylethylamine (DIPEA) for three days at room temperature (RT), leading to intermediate **5** in good yields. To reduce the reaction time, this step was also performed using a microwave reactor, which allowed us to obtain comparable amounts of **5** after 15 min. The nitro-derivative **5** was then reduced using Pd/C at RT, leading the aniline derivative **6** which was cyclized in the presence of 1,1’-carbonyldiimidazole (CDI) and 4-dimethylaminopyridine (DMAP) for two days. As well as for the first step, this reaction was also performed using a microwave reactor, obtaining comparable yield of **7** (85%) after 15 min. Then the piperidine portion linked to the 2-oxo-benzimidazole derivative **7** was deprotected from the carbamate function in acidic conditions, obtaining a quantitative amount of **8**, which was reacted with 2-bromo-4’-chloroacetophenone in presence of triethylammine (TEA) leading to compound **2**. For the synthesis of compound **1**, the tetrazolic portion was formed before the functionalization with the *p*-chloro-phenacyl group. A solventless reaction procedure was performed [19]: **7** was heated at 125 °C for 24 h under nitrogen atmosphere in the presence of trimethylsilyl azide (TMSN_3_) and tetrabutylammonium fluoride (TBAF). Treatment of the crude reaction mixture with ice and 10% HCl led to **9** as pure precipitate, whose BOC-protective group was removed by acidic hydrolysis. Then, **10** was reacted with 2-bromo-4’-chloroacetophenone in the presence of triethylamine (TEA) at 0 °C. Isolation of the desired product was performed using a selective liquid extraction and purity of **1** was assessed by HPLC. The structure of the compound was confirmed by LC-MS analysis.

### 2.2. Optimization of Analytical Procedure

#### 2.2.1. Derivatization Conditions 

**Figure 2:**
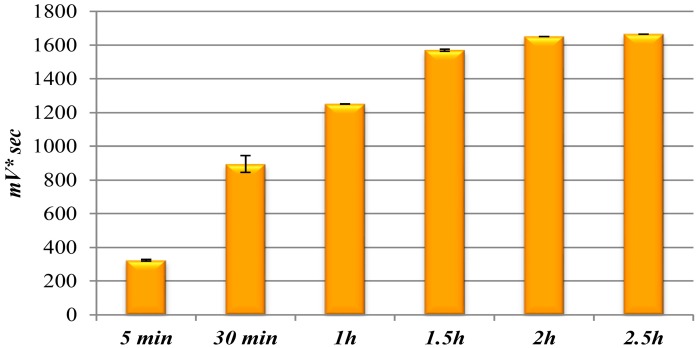
Different derivatization times of 200 nM sphinganine samples: area values obtained by analysis of triplicates. Values are expressed as mean ± SD, n = 6, from two independent experiments.

In order to optimize the derivatization conditions (generally performed at RT), we decided to gently warm (40 °C) the samples during this procedure: different concentration of sphinganine were added to the “derivatization solution” (990 μL of borate buffer (pH 10.5), 5 μL of OPA and 0.5 μL of 2-mercaptoethanol) and allowed to react for different times (5 and 30 min, 1, 1.5, 2, 2.5 h). To evaluate the formed isoindole derivatives, the chromatographic condition previously reported was applied [17], and on the basis of the chromatographic resolution, the reaction time needed to obtain a quantitative conversion of sphinganine to the isoindole derivatives was set. The area values corresponding to a 1.5 h of reaction at 40 °C had a standard deviation lower than 15%, suggesting that the reaction was completed (Figure 2).

#### 2.2.2. Chromatographic Conditions 

**Figure 3:**
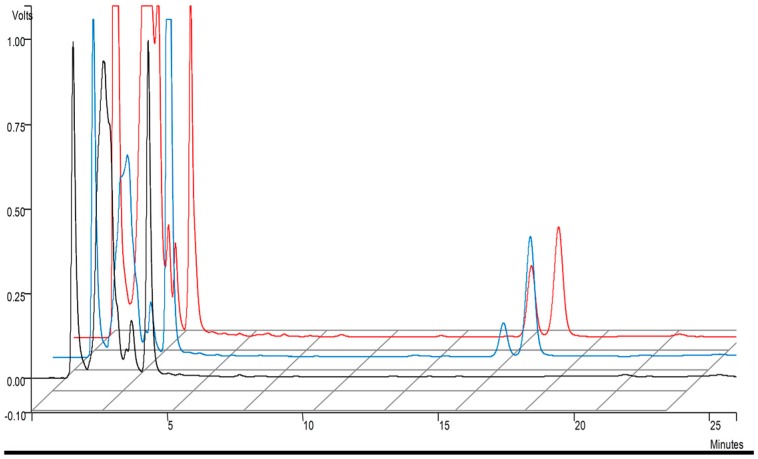
HPLC-FL chromatograms of a blank sample (black), a standard sphinganine sample (blue) and cell lysate sample after incubation (red).

The chromatographic conditions were optimized to avoid the use of phosphate buffer, in relation to a possible method application on LC-MS devices. For that reason, ammonium acetate buffer was tested in the presence of acetonitrile and/or methanol on Luna C18 ODS2 (150 × 4.6 mm, 3 μm). Detection wavelengths used were λex = 335 nm and λem = 440 nm [13]. Best results in terms of analytes resolution and retention time were achieved using a mixture of acetonitrile and ammonium acetate buffer (pH 7; 10 mM) (90−10%) at a flow rate of 0.8 mL/min (Figure 3).

#### 2.2.3. Extraction Procedure 

HEK 293 cell lysate represents a complex matrix, therefore a selective extraction procedure was needed in order to obtain a good resolution of the analytes. Cell lysate pellet (2 mg/mL) was spiked with sphinganine or 3-KDS: to 200 μL of this sample was added NaBH_4_ to reduce 3-KDS to sphinganine. The use of sphinganine-spiked sample allowed us to evaluate the complete reduction of 3-KDS. Then, 100 μL of NH_4_OH (2 M) was added and a liquid–liquid extraction procedure was performed: the best organic mixture to be used was selected by testing a different mixture of MeOH and CHCl_3_ (a mixture generally used in sphingolipids analysis). At the same time, washing of the organic phase by addition of alkaline water was tested to remove interfering lipids [20]. The data obtained highlighted that the use of alkaline solution to remove contaminates from the organic phase was necessary, and a MeOH–CHCl_3_ (2:1, *v*/*v*) mixture allowed quantitative yields to be obtained. Then, a defined volume of organic phase (150 μL of lower phase) was concentrated under N_2_ and the residue reconstituted with 145 μL of MeOH–EtOH–H_2_O (85:47.5:17.5, *v*/*v*/*v*). Then, the “derivatization solution” (5 μL) was added. Once optimized, the extraction procedure, incubation experiments of both pellet and supernatant fractions of HEK293 cell lysate (obtained after ultracentrifugation at 33,000 rpm, 40 min, 4 °C) with substrates and cofactor were performed. The data confirmed the presence of SPT activity in the pellet fraction whereas no enzyme activity was detected in the supernatant portion. 

#### 2.2.4. Method Validation 

**Figure 4:**
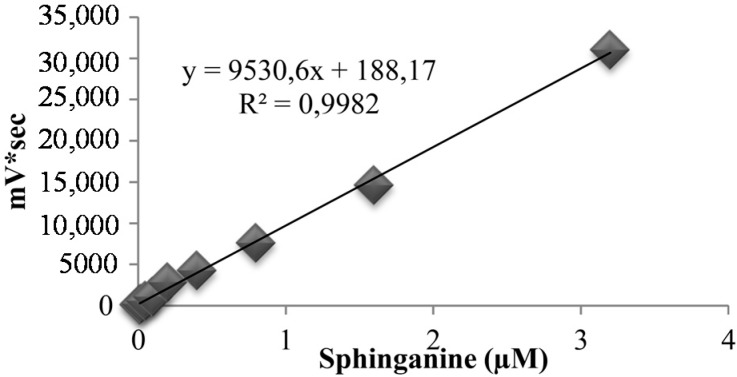
Calibration curve of sphinganine.

The method was validated according to EMEA guidelines [21]. A calibration curve was developed by analyzing blank samples spiked with sphinganine standard solutions, obtaining a linearity range from 10 nM to 3.2 μM and a correlation coefficient (R^2^) of 0.998 (Figure 4). Recovery of sphinganine was 97 ± 2% and lower limit of quantification (LLOQ) and detection (LLOD) were estimated to correspond to 10 and 5 nM, respectively. Specificity with regard to other co-eluting components was investigated by comparing the chromatograms of different batches of blank matrices to those from spiked cell lysate solutions and test samples. The chromatographic condition developed avoid the presence of an interference peak. Typical retention times for sphinganine were 16.6 ± 0.1 (*threo*) and 17.6 ± 0.2 min (*erythro*).

Low-, medium-, and high-quality control samples (QCs) were used to evaluate the accuracy and precision of the method. The data obtained were shown to be in agreement with that recommended by EMEA guidelines (Table 1) [21].

#### 2.2.5. Incubation Period Evaluation

**Figure 5:**
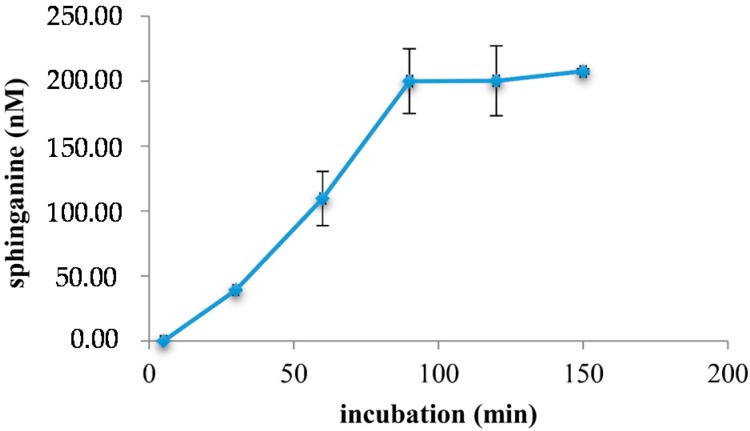
Human serine palmitoyl transferase (SPT) initial velocity evaluation.

To 2 mg/mL of cell lysate (180 μL), 10 μL of the solvent used for the control sample and 10 μL of “mix” solution containing L-serine, palmitoyl-CoA, and PLP (100 mM L-serine, 1.0 mM palmitoyl-CoA, 0.4 mM PLP) were added. Enzyme initial velocity was evaluated by incubating control samples at 37 °C for different times (5 and 30 min, 1, 1.5, 2 h) to choose the suitable incubation period. These experiments were performed by testing triplicates. Evaluation of sphinganine concentration (and therefore of the produced 3-KDS) showed a linearity of enzyme activity from 5 min to 1.5 h (Figure 5). Therefore, the selected incubation period was 1 h.

#### 2.2.6. Screening of SPT Inhibitors

Once chromatographic and assay conditions were optimized, myriocin was tested for its inhibitory activity towards SPT.

**Figure 6:**
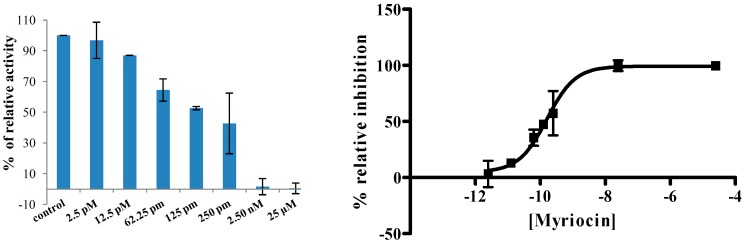
Dose-dependent inhibition of human SPT by myriocin (HPLC-FL assay).

Evaluation of dose–response inhibition of myriocin using the HPLC-FL assay was performed by testing concentrations ranging from 2.5 pM to 25 nM. The data highlighted the strong inhibitory activity of this molecule and an IC_50_ value of 160.0 ± 1.2 pM (Figure 6).

The SPT inhibitory activity of myriocin in the RIA was not assessed in this work; however, its IC_50_ value was already calculated as 0.28 nM in an analogous radiometric test [22], attesting the remarkable power of this molecule as an SPT inhibitor and the good correlation between the RIA and the HPLC-FL assay.

At the same time, compounds **1** and **2** were tested in a concentration of 50 μM by both HPLC-FL and RIA assay. The results are reported in Table 2 as a percentage of residual SPT activity. No meaningful differences between the two methods of assay were observed. In fact, compound **1** caused a percentage of residual SPT activity of 1.8 in the RIA, and of 1.3 in HPLC-FL assay; similarly, the obtained data for compound **2** in the RIA and in HPLC-FL assay were comparable (5.7% and 4.1%, respectively).

## 3. Materials and Methods

### 3.1. Chemical Synthesis

Reagents were purchased from commercial sources and used without further purification. Melting points were determined on a Kofler hot stage apparatus and were uncorrected. ^1^H-NMR and ^13^C-NMR spectra were recorded with a Varian Gemini 200 spectrometer (operating at 200 MHz). Chemical shifts (δ) are reported in parts per million related to the residual solvent signal, while coupling constants (*J*) are expressed in Hertz (Hz). Microwave-assisted reactions were run in a Biotage^®^ microwave synthesizer (Uppsala, Sweden). Merck silica gel 60 was used for flash chromatography (230−400 mesh). The chemical purity of the target compounds was determined under the following conditions: the HPLC system was an LC Workstation Prostar (Varian, Inc., Walnut Creek, CA, USA) consisting of a high-pressure mixer pump (ProStar, model 230), UV-DAD detector (ProStar, model 330) with wavelengths set at 220 and 320 nm, and a loop of 20 μL. Data were processed by a Star LC Workstation (Varian, Inc.). Chromatographic separation was performed on a Luna C18 ODS2 analytical column (150 × 4.6 mm inner diameter, 3 μm particle size, Phenomenex, Torrance, CA, USA) maintained at 25 °C. The mobile phase consisted of acetonitrile:water. The purity of each compound was >96% in either analysis. For the final products **1** and **2**, LC-MS analysis was accomplished using a PerkinElmer (Waltham, MA, USA) 200 Series micro-pump system, equipped with autosampler and column oven, both 200 Series from Perkin Elmer and an Applied Biosystems/Sciex (Foster City, CA, USA) API 4000 triple quadrupole mass spectrometer, equipped with a Turbo V electrospray ionization source (ESI).

#### 3.1.1. *tert*-Butyl 4-(2-nitro-4-cyanophenylamino)-piperidine-1-carboxylate (**5**)

In a microwave vial, DIPEA (3.5 mL, 20.0 mmol) and 4-chloro-3-nitrobenzonitrile **3** (1.83 g, 10.0 mmol) were mixed in 4 mL of dimethylformamide (DMF). Afterwards, 4-amino-1-BOC-piperidine **4** (2.40 g, 12.0 mmol) were added and the vial was capped. Reaction conditions: 15 min; 130 °C; 5 bar. After cooling, 50 mL of ethyl acetate and 10 mL of tetrahydrofuran (THF) were added to the reaction mixture. The organic layer was washed with 0.5 M solution of hydrochloric acid (2 × 40 mL) and brine in a separating funnel. The organic layer was dried over magnesium sulfate, filtrated and evaporated in vacuo, leading to the desired compound as a yellow powder (3.11 g, 9.0 mmol). Yield: 90%. The titled compound was crystallized from toluene. ^1^H-NMR (CDCl_3_, 200 MHz): 8.54 (d, 1H, *J* = 1.9 Hz), 8.45 (bs, 1H), 7.61 (dd, 1H, *J* = 8.8 Hz, *J* = 1.8 Hz), 6.96 (d, 1H, *J* = 8.8 Hz), 4.11–3.88 (m, 2H), 3.74-3.65 (m, 1H), 3.11–2.98 (m, 2H), 2.19-1.98 (m, 2H), 1.96–1.58 (m, 2H), 1.48 (s, 9H).

#### 3.1.2. *tert*-Butyl 4-(2-amino-4-cyanophenylamino)-1-piperidinecarboxylate (**6**)

*tert*-butyl 4-(2-nitro-4-cyanophenylamino)-1-piperidinecarboxylate (**5**) (1.21 g, 3.5 mmol) was dissolved in 120 mL of a solution of THF–absolute EtOH (5:95, *v*/*v*). To the obtained solution, Pd/C was added, and the mixture was placed under hydrogen and stirred at RT for 4 h. Afterwards, the solution was filtered on a silica pad and washed with ethyl acetate (100 mL). The organic phase was concentrated in vacuo, leading to a white powder (0.996 g, 3.15 mmol). Yield: 90%. The titled compound was crystallized from isopropanol. ^1^H-NMR (CDCl_3_, 200 MHz): 7.15 (d, 1H, *J* = 8.2 Hz), 6.96 (s, 1H), 6.60 (d, 1H, *J* = 8.2 Hz), 4.11–3.92 (m, 3H), 3.57-3.39 (m, 3H), 3.02–2.86 (m, 2H), 2.08–2.01 (m, 2H), 1.47 (s, 11H). ^13^C-NMR (CDCl_3_): 154.91, 141.22, 133.34, 126.78, 120.70, 120.10, 110.51, 99.49, 80.04, 49.93, 42.77, 32.36, 28.73.

#### 3.1.3. *tert*-Butyl 4-(6-cyano-2-oxobenzimidazol-3-yl)piperidine-1-carboxylate (**7**)

Method A: to a solution of **6** (0.65 g, 2.0 mmol) in 25 mL of anhydrous THF, CDI (0.68 g, 4.2 mmol), and DMAP (0.24 g, 2.0 mmol) were added and the resulting mixture was heated at 100 °C for 5 h. The solution was cooled to RT and allowed to sit for two days. Then, it was diluted with 80 mL of AcOEt and washed with aqueous hydrogen chloride (0.6 M, 3 × 60 mL) and brine (2 × 60 mL), dried with magnesium sulfate, filtered, and concentrated. The crude product was purified by flash chromatography (AcOEt:*n*-hexane 3:1), dissolving the mixture in CHCl_3_ (10 mL), or by crystallization (toluene) (0.623 g,1.82 mmol). Yield: 91%. Method B: compound **6** (0.65 g, 2.0 mmol), *N*,*N*’-carbonyldiimidazole (0.68 g, 4.2 mmol), DMAP (0.24 mg, 2.0 mmol), and anhydrous THF (10 mL) were mixed in a microwave vial (1–20 mL). The mixture was heated at 120 °C, for 15 min (7 bar; cooling on; stirring on). The mixture obtained was diluted with 80 mL of AcOEt and washed with aqueous hydrogen chloride (0.6 M, 3 × 60 mL) and brine (2 × 60 mL), dried with magnesium sulfate, filtered, and concentrated. The crude product was purified by flash chromatography (AcOEt:*n*-hexane 3:1), dissolving the mixture in CHCl_3_ (10 mL), or by crystallization (toluene) (0.582 g, 1.70 mmol). Yield: 85%. ^1^H-NMR (DMSO, 200 MHz): δ 11.36 (bs, 1H), 7.49–7.36 (m, 3H), 4.48–4.23 (m, 1H), 4.23–4.05 (m, 2H), 2.89–2.73 (m, 2H), 2.32–2.16 (m, 2H), 1.74–1.65 (m, 2H), 1.43 (s, 9H). ^13^C-NMR (DMSO): δ 153.13, 153.00, 132.46, 127.91, 125.02, 119.01, 110.99, 101.80, 108.47, 78.22, 49.91, 42.44, 27.84, 27.55.

#### 3.1.4. 2,3-Dihydro-2-oxo-1-(piperidin-4-yl)-benzimidazole-5-carbonitrile (**8**)

CF_3_COOH (1.15 mL, 15.0 mmol) was added to a suspension of **7** (0.51 g, 1.5 mmol) in 5 mL of CH_2_Cl_2_. The obtained solution was stirred at RT for 2 h, then concentrated in vacuo and a saturated solution of NaHCO_3_ was added to reach pH 6–7. Then, the mixture was put in an ice bath for 1 h. The obtained precipitate was filtered and dried at 50 °C in vacuo (0.290 g, 1.2 mmol). Yield: 80%. ^1^H-NMR (DMSO, 200 MHz): δ 11.50 (bs, 1H), 9.25 (br, 1H), 7.69 (d, 1H, *J* = 8.0 Hz), 7.53–7.41 (m, 2H), 4.63–4.56 (m, 1H), 3.47–3.01 (m, 4H), 2.68–2.52 (m, 2H), 1.97–185 (m, 2H). ^13^C-NMR (DMSO): δ 152.88, 132.08, 128.06, 124.98, 118.93, 111.27, 108.38, 102.1, 46.79, 42.42, 24.64. 

#### 3.1.5. {1-[2-(4-Chlorophenyl)-2-oxoethyl]piperidin-4-yl}-2,3-dihydro-2-oxo-benzimidazole-5-carbonitrile (**2**)

Et_3_N (167 μL, 1.2 mmol) and 2-bromo-*p*-chloroacetophenone (1.2 mmol) were added to a solution of **8** (0.24 g, 1.0 mmol) in 6 mL of THF–anhydrous DMF (1:1, *v*/*v*), and the resulting mixture was stirred at RT for 2 h. The reaction mixture was then concentrated, treated with ice/water, and extracted with CHCl_3_. The organic layer was dried with magnesium sulfate, filtered, and concentrated in vacuo. The crude mixture was purified by crystallization or flash chromatography (0.276 g, 0.70 mmol). Yield: 70%. ^1^H-NMR (DMSO, 200 MHz): δ 11.44 (bs, 1H), 8.02–.19 (m, 7H), 5.05 (s, 2H), 4.66–4.55 (m, 1H), 3.73–3.60 (m, 2H), 2.87–2.63 (m, 2H), 2.05–1.81 (m, 4H). ^13^C-NMR (DMSO): δ 190.06, 152.88, 139.04, 131.92, 129.48, 128.57, 128.03, 125.4, 118.94, 111.27, 108.70, 102.15, 60.74, 52.24, 47.99, 46.30, 24.82.

#### 3.1.6. *tert*-Butyl-4-[2-oxo-5-(tetrazol-5-yl)-2,3-dihydro-benzimidazol-1-yl]piperidine-1-carboxylate (**9**)

In a screw capped vial equipped with a magnetic stir bar, TBAF (1 M in THF, 0.5 mL, 0.5 mmol), *tert*-butyl 4-(6-cyano-2-oxobenzimidazol-3-yl)piperidine-1-carboxylate (**7**) (0.34 g, 1.0 mmol), and TMSN_3_ (0.26 mL, 2.0 mmol) were added under nitrogen. The resulting mixture was heated under vigorous stirring at 125 °C for 48 h. After cooling, the crude reaction mixture was treated with ice and 10% HCl aqueous solution, obtaining a suspension. The solid was filtered and dried at 50 °C in vacuo (0.289 g, 0.75 mmol). Yield: 75%. ^1^H-NMR (DMSO, 200 MHz): δ 11.22 (bs, 1H), 7.75–7.36 (m, 3H), 4.32 (m, 1H), 4.08–4.04 (m, 2H), 2.92–2.73 (m, 2H), 2.19–2.12 (m, 2H), 1.72–1.59 (m, 2H), 1.38 (s, 9H). ^13^C-NMR (DMSO): δ 153, 10, 131.00, 128.24, 119.21, 115.57, 108.39, 106.34, 78.21, 55.32, 49.67, 42.41, 27.88, 27.51.

#### 3.1.7. 1-(Piperidin-4-yl)-5-(tetrazol-5-yl)-1H-benzimidazol-2-one (**10**)

CF_3_COOH (0.54 mL, 7.0 mmol) was added to a suspension of **9** (0.270 g, 0.7 mmol) in 5 mL of CH_2_Cl_2_. The obtained solution was stirred at RT for 2 h and concentrated in vacuo. Ice and a saturated solution of NaHCO_3_ were added to reach pH 6–7. The resulting precipitate was filtered and dried at 50 °C in vacuo (0.140 g, 0.49 mmol). Yield: 70%. ^1^H-NMR (DMSO, 200 MHz): δ 10.92 (bs, 1H), 7.80–7.25 (m, 3H), 4.56–4.48 (m, 1H), 3.27–1.65 (m, 9H). ^13^C-NMR (CD_3_OD): δ 165.73, 156.21, 132.10, 122.56, 122.088, 121.03, 110.71, 108.56, 52.08, 46.61, 29.57.

#### 3.1.8. {1-[2-(4-Chlorophenyl)-2-oxoethyl]piperidin-4-yl}-5-(2H-tetrazol-5-yl)-2,3-dihydro-benzimidazol-2-one (**1**)

Et_3_N (0.07 mL, 0.5 mmol) was added to an ice bath-cooled solution of **10** (0.143 g, 0.5 mmol) in 3 mL of anhydrous DMF. A solution of 2-bromo-*p*-chloroacetophenone (0.117 g, 0.5 mmol) in DMF (0.5 mL) was added dropwise. The reaction was stirred at 0 °C for 1.5 h. Then, ice was added to the reaction mixture and the formed precipitate was filtered and triturated with diethyl ether, obtaining a white residue (0.123 g, 0.28 mmol). Yield: 56%. The white residue was treated with a small amount of MeOH and each organic fraction was analyzed with a HPLC system coupled to a detector UV set at 254 nm, to assess fraction purity. The organic fractions were collected and dried under nitrogen. Chromatographic separation was performed on a Luna C18 ODS2 analytical columns (150 × 4.6 mm inner diameter, 3 μ particle size, Phenomenex) maintained at 25 °C. The mobile phase consisted of acetonitrile-buffer (10 mM AcONH_4_, adjusted to pH 7.0 with NH_4_OH; 50:50, *v*/*v*) at a flow rate of 0.5 mL/min. ^1^H-NMR (DMSO, 200 MHz): δ 11.11 (bs, 1H), 8.07–7.34 (m, 7H), 6.65 (s, 2H), 4.43–3.95 (m, 2H), 3.83 (s, 1H), 3.23–3.01 (m, 2H), 2.45–1.18 (m, 4H). ^13^C-NMR (DMSO): δ 191.66, 155.88, 132.60, 132.01, 130.57, 129.74, 128.57, 128.90, 128.6, 126.2, 109.97, 108.66, 60.74, 53.24, 48.92, 36.30.

### 3.2. Radiometric Inhibition Assay (RIA)

#### 3.2.1. Materials

Pyridoxal-5’-phosphate, L-Serine, and palmitoyl coenzyme A were purchased from Sigma-Aldrich (St. Louis, MI, USA). Sphingosine-1-phosphate (S1P) was purchased from Avanti Polar Lipids. DMEM culture media and fetal bovine serum (FBS) were purchased from EuroClone Life Science Division (Milan, Italy). l-[^3^H(G)]-Serine was purchased from PerkinElmer, Inc. (Wellesley, MA, USA).

Human breast cancer cell line MDA-MB-231, from America Type Culture Collection (ATCC), Rockville, MD, USA, was chosen as the source of serine-palmitoyl transferase (SPT) enzyme. Cells were maintained at 5% CO_2_, 95% humidity at 37 °C in DMEM, supplemented with 10% FBS and 1% penicillin/streptomycin solution as antibiotics. Cells were seeded in 150 mm Petri dishes (3 × 106 cells in 24 mL) and left to grow for at least 3 days, until 80–90% confluence was reached, before being collected for microsomal membranes fraction extraction, due to the localization of the studied enzyme at this level.

#### 3.2.2. Radiometric Inhibition Assay (RIA) Procedure

Microsomal membranes fraction was collected through MDA-MB-231 homogenization in a specific extraction buffer (Hepes pH 7.4, 25 mM; EGTA pH 7.8, 5 mM; NaF 50 mM; leupeptin 10 μg/mL and tripsin inhibitor 10 μg/mL) and centrifugation at 4070 rpm for 10 min at 4 °C. After Bradford protein assay, the enzymatic assay was performed by incubating 300 μg of total proteins at 37 °C for 30 min in the reacting buffer (Hepes pH 8.3, 100 mM, EDTA pH 7.4, 2.5 mM and DTT 5 mM) containing pyridoxal-5’-phosphate 50 μM as enzymatic cofactor and l-[^3^H(G)]-Serine (1 mM, 2 μCi specific activity 26 Ci/mmol), l-Serine (1 mM), and palmitoyl coenzyme A (200 μM) as substrates. Counts per minute (CPM) were measured with β-counter (Packard Tri-Carb Liquid Scintillation Analyzer, model 2900TR, GMI Inc., Ramsey, MI, USA) after washing the organic phase three times with bi-distilled water, dividing the two phases (hydrophilic and organic) with various centrifugations, drying up the samples overnight at 39 °C, and re-suspending them in scintillation cocktail. Results are expressed as a percentage of enzymatic activity with respect to the control (DMSO) ± standard deviation.

### 3.3. HPLC-FL assay

#### 3.3.1. Materials

d,l-*erythro*-sphinganine (*erythro* 77%; *threo* 23%) and 3-keto-dihydrosphingosine hydrochloride (3-KDS) powders (>98.0% purity) were supplied from Matreya LLC^®^ (Pleasant Gap, PA, USA). l-Serine, pyridoxal 5’-phosphate monohydrate (PLP), and palmitoyl coenzyme A lithium salt (≥90%) Bioreagent grade were purchased from Sigma-Aldrich (St. Louis, MI, USA). Dimethylsulphoxide (DMSO), absolute ethanol, *O*-phthalaldehyde (OPA), 2-mercaptoethanol, EDTA, sodium borohydride (NaBH_4_), HEPES, and ammonium hydroxide solution were purchased from Sigma-Aldrich (St. Louis, MI, USA). HPLC grade acetonitrile (ACN), methanol (MeOH), chloroform (CHCl_3_), and ammonium acetate (AcONH_4_) were purchased from Sigma-Aldrich (St. Louis, MI, USA). Deionised water was produced by a Milli-Q Millipore Water System (Millipore, MA, USA). All the other reagents and materials were of analytical grade and supplied from commercial sources. The aqueous and organic components of the mobile phase, degassed under pressure, were mixed by the HPLC. The LC mobile phases were filtered through 0.2 μm cellulose acetate membrane filters (Sartorius Stedim Biotech S.A., Aubagne Cedex, France) with a solvent filtration apparatus. 

Standard solutions: singular stock solutions of d,l-*erythro*-sphinganine and 3-KDS in MeOH were prepared, each with a concentration of 1.0 mM, by using volumetric flasks; these were stored at −20 °C. To obtain a final concentration of 100 μM, appropriate dilutions of stock standard solutions were prepared by diluting 1 mL of each solution to 10 mL. Successively, these solutions were diluted in glass tubes (10 mL), to reach final concentrations of 10, 1 and 0.1 μM. These were stored at −20 °C.

Stock solutions: borate buffer (pH 10.5; 3% *p*/*v*): 3.00 g of boric acid were dissolved in milliQ water. NaOH was added to reach pH = 10.5. The solution was brought to volume using a 100 mL volumetric flask. The buffer was stored at 3 °C. OPA (*O*-phthalaldehyde): 50 mg of OPA were dissolved in 1 mL of EtOH. The solution was stored in an amber glass vial at 3 °C (1 month maximum). EDTA (pH 8; 0.5 M): 1.86 g of EDTA were dissolved in milliQ water and pH is adjusted by addition of NaOH. The solution was brought to volume in a 50 mL volumetric flask. HEPES buffer (pH 8.0; 0.5 M): 5.95 g of HEPES was dissolved in milliQ water and pH was adjusted by addition of NaOH. The solution was brought to volume in a 50 mL volumetric flask. Lysis buffer: 1 mL of HEPES buffer and 20 mL of EDTA were mixed and brought to volume in a 10 mL volumetric flask. An amount of 200 mM L-Serine: 210.18 mg of L-Serine was dissolved in milliQ H_2_O and brought to volume in a 10 mL volumetric flask. An amount of 5 mM PLP: 61.785 mg of PLP was dissolved in milliQ H_2_O and brought to volume in a 50 mL volumetric flask. Palmitoyl-CoA (lithium salt; 5 mM): 5.0 mg of palmitoyl coenzyme A lithium salt was dissolved in 1 mL of milliQ H_2_O.

Freshly prepared solutions: “derivatization solution”: in 1.5 mL snap cap vial, 990 μL of borate buffer (pH 10.5; 0.5 mM), 10 μL of OPA (50 mg/mL in ethanol), and 0.5 μL of 2-mercaptoethanol were mixed. MeOH/EtOH/H_2_O (85:47.5:17.5, *v*/*v*/*v*) solution: in a 15 mL falcon tube, 3.40 mL of MeOH, 1.9 mL of EtOH, and 0.7 mL of milliQ H_2_O were mixed. MeOH/CHCl_3_ (2:1, *v*/*v*): in a 15 mL falcon tube, 10 mL of MeOH, and 5 mL of CHCl_3_ were mixed. Sodium borohydride (NaBH_4_) solution: about 6 mg of sodium borohydride was dissolved in 1 mL of milliQ water. Substrates mix: to 125 μL of l-Serine were added 20 μL of PLP, 50 μL of palmitoyl-CoA and 55 μL of milliQ H_2_O.

#### 3.3.2. HPLC-FL Assay Procedure

The HPLC system was an LC Workstation Prostar (Varian, Inc., Walnut Creek, CA, USA) consisting of a high-pressure mixer pump (ProStar, model 230), CTO-10Avp column oven, spectrofluorometric detector (ProStar, model 363), and a loop of 50 μL. Data were processed by a Star LC Workstation (Varian, Inc.). Chromatographic separation assay was performed by a Luna C18 ODS2 analytical columns (150 × 4.6 mm inner diameter, 3 μm particle size, Phenomenex, Torrance, CA, USA) maintained at 25 °C. The mobile phase consisted of acetonitrile-buffer (10 mM AcONH_4_, adjusted to pH 7.0 with NH_4_OH) (90:10, *v*/*v*) at a flow rate of 0.8 mL/min. Excitation and emission wavelengths were set at 335 and 440 nm, respectively. The analyses were carried out in isocratic mode. Results are expressed as a percentage of enzymatic activity with respect to the control (vehicle) ± standard deviation.

### 3.4. Cell Culture and SPT Cell Lysate Preparation

Human embryonic kidney cells (HEK293) were grown in DMEM-F12 medium. Cultures were maintained at 37 °C in a 5% CO_2_ atmosphere at 100% humidity. Cell membrane microsomes were prepared as follows: a HEK293 cell monolayer in a 10 cm dish was washed with 5 mL of PBS and mechanically removed through a scraper. Then, the petri dish was washed with 5 mL of PBS. The cell suspension was collected and centrifuged for 5 min at 1200 rpm. Then, the pellet was suspended in 500 μL lysis buffer (pH 8; 50 mM HEPES and 1 mM EDTA). The cell suspension was sonicated for 15 s at 50% power and 50% pulsation (Sonopuls HD 2070; Bandelin, Berlin, Germany) and centrifuged at 2500 rpm at 4 °C for 30 min. The supernatant was then centrifuged at 33,000 rpm for 40 min at 4 °C. The pellet was re-suspended in the lysis buffer: protein determination was performed by colorimetric Bradford assay, using the protein assay kit from Bio-Rad (Hercules, CA, USA). Cell lysate was diluted to reach a concentration of ≈2 mg/mL and used to evaluated SPT activity.

### 3.5. Incubation and Sample Extraction Procedure

The procedure was performed in a 1.5 mL snap cap polypropylene vial. To a 180 μL aliquot of cell lysate (≈2 mg/mL) was added 10 μL of inhibitor solution or vehicle (control sample) and 10 μL of “substrate mix” (l-serine, PLP, palmitoyl-CoA). Samples were incubated at 37 °C for 1 h. Experiments were performed in triplicate. To ensure the absence of interferences, a background analysis of each cell lysate batch was carried out. After incubation, 50 μL of a NaBH_4_ aqueous solution (≈6 mg/mL) was added to samples and left to react at RT for 5 min. Then, 100 μL of NH_4_OH (2 M) was added. Each sample was vortexed for 10 s; then, 600 μL of MeOH/CHCl_3_ (2:1, *v*/*v*) was added and the sample vortexed again (30 s). Once the organic and aqueous phase separation was achieved by centrifugation (14,000 rpm, 1 min), 200 μL of the supernatant was removed and 600 μL of NH_4_OH (2 mM) was added. The sample was vortexed for 30 s and centrifuged at 14,000 rpm for 1 min. An amount of 150 μL of the organic phase was collected and evaporated under a gentle stream of nitrogen in an amber glass vial. The residue was reconstituted with 145 μL of MeOH/EtOH/H_2_O (85:47.5:17.5, *v*/*v*/*v*) and 5 μL of “derivatization solution” was added. Derivatization reaction was performed at 40 °C for 2 h. Then, 50 μL of this latter solution was injected onto the HPLC-FL. SPT initial velocity was evaluated by performing different incubation periods (5, 30, 60, 90, 120 and 150 min) at 37 °C. Each incubation period was tested in triplicate. Subsequently, the selected compounds were tested in HPLC-FL in triplicate.

### 3.6. Bioanalytical Method Validation

The described method was validated in terms of linearity, lower limit of detection and quantification (LLOD and LLOQ), recovery, specificity, stability, precision, and trueness according to international guidelines on the bioanalytical method validation. Calibration curves were obtained by spiking the blank matrix with a known concentration of d,l-*erythro*-sphinganine to provide concentrations of 0.010, 0.020, 0.050, 0.075, 0.10, 0.20, 0.40, 0.80, 1.60 and 3.20 μM. The calibration curves of peak area versus concentration of d,l-*erythro*-sphinganine were built. Least squares regression parameters for the calibration curve were calculated, and the concentrations of the test samples were interpolated from the regression parameters. Sample concentrations were determined by linear regression, using the formula Y = mX + b, where Y = peak area, X = concentration of the standard, m = the slope of the curve and b = the intercept with y-axis. Correlation coefficient for the calibration curves was >0.99. Accuracy and within-run and between-run precision were assessed on quality control samples (QC samples) and determined by replicate analysis using seven determinations of different concentration levels: Low QC (100 nM), medium QC (800 nM), and high QC (1600 nM). When unknown samples were assayed, a control and a fortified blank sample were processed simultaneously for quality control. LLOD and LLOQ were determined as analyte concentrations giving signal-to-noise ratios of 3 and 10, respectively. All the analyses were conducted using GraphPad InStat (GraphPad Software, Inc., La Jolla, CA, USA).

## 4. Conclusions

In this work, we described the application and validation of an HPLC-FL-based method to screen the SPT-inhibitory activity of two heterocyclic compounds reported in a patent [18] and which were re-synthesized in our research laboratories. Different steps of method development and optimization were pursued: in a first stage, analytical conditions were developed by evaluating the derivatization and chromatographic condition, extraction procedure, and method validation according to EMEA guidelines. After optimization of the analytical conditions, the assay procedure was evaluated in relation to enzyme initial velocity. Once fully optimized, the method was applied to assess the SPT-inhibitory activity of the above-mentioned derivatives and of the reference SPT-inhibitor myriocin. The obtained results showed a good correlation between the reference RIA method and the HPLC-FL method. So, the improvements introduced in the analytical procedure made this non-radiometric method easily applicable in medicinal chemistry labs for a preliminary and rapid screening of potential SPT-inhibitors characterized by diverse chemical structures.

## Figures and Tables

**Table 1:** Accuracy and precision evaluation (RSD %) of quality control (QC) samples.

QC Sample	Accuracy	Precision	
		Within-Run	Between-Run
LOW (100 nM)	9.92%	9.1%	13.3%
MEDIUM (800 nM)	5.73%	14.8%	11.0%
HIGH (1600 nM)	8.57%	6.3%	7.4%

**Table 2:** Screening of compounds **1** and **2** (at a concentration of 50 μM) on human SPT by RIA and HPLC-FL assay. ^1^

Compound	% of Residual	SPT Activity
	RIA	HPLC-FL
1	1.8 ± 0.8	1.3 ± 0.5
2	5.7 ± 2.1	4.1 ± 1.5

^1^ Data are obtained from three experiments and values are expressed as average ± SD.
